# Sustainable control and integrated management through a one health approach to mitigate vector-borne disease

**DOI:** 10.1016/j.onehlt.2025.101018

**Published:** 2025-03-28

**Authors:** Jing Ni, Jinna Wang, Wenrong Zhang, Enfu Chen, Yi Gao, Jimin Sun, Wenzhong Huang, Jun Xia, Weiming Zeng, Junxiang Guo, Zhenyu Gong

**Affiliations:** a School of Public Health, Hangzhou Medical College, Hangzhou 310053, China; b Department of Communicable Disease Control and Prevention, Zhejiang Provincial Center for Disease Control and Prevention, Hangzhou 310051, China; c Zhejiang Patriotic Health Development Center, Hangzhou 310006, China; d Pujiang Center for Disease Control and Prevention, Pujiang 322200, China; e Longyou Centre for Disease Control and Prevention, Quzhou 324400, China

**Keywords:** Mosquito-free village, Four pest-free village, One health, Global vector control response 2017–2030 (GVCR), Vector-borne disease, China, SPRP

## Abstract

Vector-borne diseases have a huge negative impact globally. WHO has proposed the concepts of “sustainable control” and “integrated management” to control vector organisms. It formally proposed the “Global Response to Vector Control 2017-2030” in 2017. Combining this concept with the One Health (OH) concept, China has proposed a scalable approach to ----- Four Pest-Free village, aiming to reduce the incidence of vector-borne diseases by reducing vector density through the use of environmentally friendly treatments, thereby reducing the incidence of vector-borne diseases and achieving human, animal and environmental health. The results of the study show that the “Four Pest-Free Village” has a significant effect on controlling vector populations, transforming the rural landscape, increasing the area of wetlands and reducing the incidence of vector-borne diseases.

The response of Zhejiang Province's Four Pest-Free Village to vector-borne diseases, particularly mosquito-borne diseases, provides a proof of concept that a multisectoral effort involving community members with the concept of One Health and the GVCR as proposed by the WHO can be effective in responding to outbreaks of zoonotic diseases while also protecting the national economy and reducing national financial support for infectious diseases.

## 1 Introduction

After the COVID-19 pandemic in 2019, insect-borne diseases such as malaria and dengue fever have received renewed global attention. The increasing number of cases of arboviral diseases due to climate and environmental changes has been a concern. An estimated 4 billion people worldwide are at risk of infection from arboviruses, and this number is estimated to increase to 5 billion by 2050 [1]. In December 2023, the World Health Organization (WHO) set the current global dengue surge at level 3, the highest WHO emergency level, to support countries in strengthening surveillance capacity and implementing response activities. By the end of August 2024, there were more than 12.3 million cases of dengue fever globally, almost double the 6.5 million cases reported in 2023. Existing anti-mosquito technologies are not able to achieve long-term mosquito control due to their high price, the development of resistance in mosquitoes, and the long cycle of vaccine development, and the technology for complete elimination of mosquitoes has not yet emerged.

In May 2017, the 70th World Health Assembly adopted the Global Vector Control Response (GVCR) 2017–2030 [2], which aims to achieve a vector-free world through two core elements: increasing capacity and capability in vector control, and strengthening basic and applied research and innovation. With “strengthening inter- and intra-sectoral action and collaboration”, “engaging and mobilizing communities”, “enhancing monitoring and evaluation of vector surveillance and interventions” and “Promoting and integrating tools and methods” as the four pillars that work together to reduce the burden and threat of vector-borne infections through effective and sustainable vector control adapted to local conditions. On October 2, 2024, the World Health Organization (WHO) launched the Global Strategic Preparedness, Readiness and Response Plan (SPRP), which is aligned with the Global Vector Control Response 2017–2030 and the Global Arbovirus Initiative launched in 2022 conceptually aligned to respond to the global pandemic of arboviruses, aiming to reduce the burden of disease, suffering and death caused by arboviral diseases by promoting a coordinated global response.

In recent years, significant progress has been made in vector control and disease surveillance in a lot of regions, aligning closely with the action pillars of the Global Vector Control Response (GVCR).In island and regions, such as Réunion Island and Mayotte in France, the French Agency for Food, Environmental and Occupational Health & Safety (Anses) has established multidisciplinary working groups to develop a “One Health” approach for monitoring and evaluating Integrated Vector Management Systems (IVMS) [3].This approach reflects the action pillars of the GVCR and emphasizes the close interconnection between human, animal, and environmental health.In India, an innovative vector control strategy called “Incredible paint” is being promoted, aligning with the GVCR's “Promotion and Integration of Tools” pillar by enhancing sustainability and reducing environmental impact. [4].Early vector surveillance is underway in some African countries, particularly in settings where there is usually less of concern, such as cemeteries, to reduce the incidence of urban malaria and other vector-borne diseases [5]. From 2018 to 2019, a one-year community-based study in Selangor, Malaysia, used gravid mosquito traps (GOS traps) and dengue NS1 antigen testing to monitor dengue/aedes mosquitoes indoors and outdoors, exemplifying the GVCR initiative [6]. Singapore's dengue control program, which includes source reduction, interdepartmental collaboration, community engagement, and robust monitoring, serves as a successful GVCR model [7]. China has also made significant progress in vector control and disease surveillance. Four Pest-free village demonstrate “sustainable vector control measures” and “engaged and mobilized communities” in Zhejiang Province. This study presents the results of anti-mosquito (including synchronized control of cockroaches, flies and rodents)implemented since 2016.These experiences, including innovative measures to address climate change and resource constraints, offer valuable insights for other regions facing vector-borne disease challenges.

## 2 Materials and methods

### 2.1 Study area

The study area (Zhejiang Province, China) is located in the southeastern coastal region of China, which has a subtropical monsoon climate with a significant monsoon, abundant rainfall, synchronized rain and heat seasonal changes, a diverse allocation of climatic resources, and numerous meteorological disasters. Due to the (average annual temperature of 15–18 °C, January and July are the lowest and highest temperature of the year, respectively, May and June for the concentrated rainfall period. The average annual rainfall is 1105 to 2047 mm) [8].Therefore, it is very easy for mosquitoes to breed. Since ancient times, mosquito-borne diseases have been rampant in Zhejiang Province in the summer after typhoons. Since the inception of the Patriotic Hygiene Campaign, Zhejiang Province has been seeking long-term effective and sustainable vector control methods.

### 2.2 Definitions

Four pest-free villages(or FPFV), also known as Sustainable Vector Management Villages (SVMV),are villages through social mobilization, health education and promotion, environmental rehabilitation and facility renovation, effective management of the countryside vector (mosquitoes, flies, cockroaches, rodents) breeding place and habitats, and apply physical and biological and other comprehensive preventive measures, so that the biological density of the vector is controlled at a certain level. According to the four stages of controlling the vector, they are called “mosquito-free village(or MFV) and flies-free village(or FFV)”, “mosquito and fly-free village(or MMFV)”, “four pest-free village(or FPFV)” and “ mosquito free-community and campus(or MFCC) ” respectively.

### 2.3 Specific method

Four pest-free villages have established village-level organizations, trained village cadres and key members, including party members, cadres and volunteer teams, carried out extensive social mobilization and health education, and, in conjunction with the construction of village-level systems, mobilized community members to institutionalize, normalize and make the construction of “SVMV” permanent. Based on the national standards related to the four pests, the pilot areas explored in practice the level of hazard control achievable in the countryside, and established standards and evaluation systems for the creation of “SVMV”. Environmental management is mainly carried out through environmental modification, physical measures and biological measures, with less application for chemical measures as the four pest-free village adheres to the concept of great health and sustainable vector control.

Environmental modification: Mosquitoes spend three stages of their lives in water, by removing stagnant water in mosquito breeding grounds or keeping the water flowing can effectively reduce the number of mosquitoes. This can be done by (i) filling potholes in the ground and removing small storage containers such as broken cans and bowls; (ii) properly storing used tires and water tanks with large storage capacity; (iii) removing stagnant water from ditches; (iv) keeping sewers clear; and (v) indirect irrigation. Rodent densities are strongly correlated with environmental carrying capacity: a limited environment can only provide resources for the survival of a limited number of organisms. Four pest-free villages reduce the source of rodents by removing garbage and properly storing food, and reduce the habitat of rodents by blocking rodent holes and hardening road surfaces, so as to ultimately achieve the goal of reducing the environmental capacity of rodents. The problem of fly breeding can be solved by controlling and managing the breeding places of flies, mainly by separating and disposing of household garbage in residential areas, farmers' markets and large supermarkets.

Physical measures: Commonly used in the control of rodent density, mainly using the rodent blocking boards, rodent-proof fences and rodent-proof nets. At the same time, mosquitoes, flies, rats and cockroaches are eliminated by using sticky traps or light trapping(solar mosquito lamp).

Biological measures: Four pest-free village of biological defense is mainly used in the control of mosquitoes, the application of mosquitoes natural enemies of fish [9] to deal with mosquito larvae, Qingtian County, Zhejiang Province, rice paddies to raise loach in the fight against mosquitoes played a very good effect.

The above three measures are usually used in combination, which reduces the number of vectorial organisms in four pest-free villages, also establishes a harmonious ecosystem.

At the same time, with reference to the “Methods for monitoring the density of vector organisms-Mosquitoes” [10], “Methods for monitoring the density of vector organisms-Flies” (GB/T 23796–2009) [11], “Methods for monitoring the density of vector organisms-Rodents” (GB/T 23798–2009) [12], “Methods for monitoring the density of vector organisms-Blattella” (GB/T 23795–2009) [13], respectively, we used trap light method, breteau index method, cage trapping method, visual inspection method, sticky trap method and sticky rodent method. (GB/T 23796–2009) [11], “Vector Density Monitoring Methods for Rodents” (GB/T 23798–2009) [12] and “Vector Density Monitoring Methods for Blatted Cockroaches” (GB/T 23795–2009) [13]. The mosquitos, flies, cockroaches and rodents densities were surveyed by mosquito trapping lamps, breteau index method, cage baiting method, visual inspection method, sticky trapping method, sticky rodent boards method/clamping (cages) at night, rodent tracking method, and path index method, respectively. Refer to “Vector Density Control Level of Mosquitoes” (GB/T 27771–2011) [14], “Vector Density Control Level of Flies” (GB/T 27772–2011) [15], “Vector Density Control Level of Gossip Cockroaches” (GB/T 27773–2011) [16], “Vector Density Control Levels of Rodents” (GB/T 27770–2011) [17], and classify mosquitoes, flies, cockroaches and rodents, and study the establishment of a group standard suitable for country areas.

### 2.4 Statistical analysis

Descriptive statistics were used to summarize the change in mosquito density of the first demonstration village of four pest-free villages from 2016 to 2023, and to present visualization of provincial demonstration villages in Zhejiang Province in 2021–2022. At the same time, these 250 provincial demonstration villages were sampled and evaluated, 38 villages were randomly selected, the questionnaire included nine parts, and the density control part had the lowest score rate. Data analysis was performed using Microsoft Excel (version 2019, Microsoft, Redmond, USA),R studio4.4.1,and visualization analysis was performed using ARCGIS10.8.

#### 2.4.1 Study results

*Practical history of countryside sustainable vector management in Zhejiang province(ZJCSVM).*

#### 2.4.2 Four Pest-Free village1.0

(1) *Mosquito-Free village(MFV) and Fly-Free village(FFV):*

**Fig. 1:**
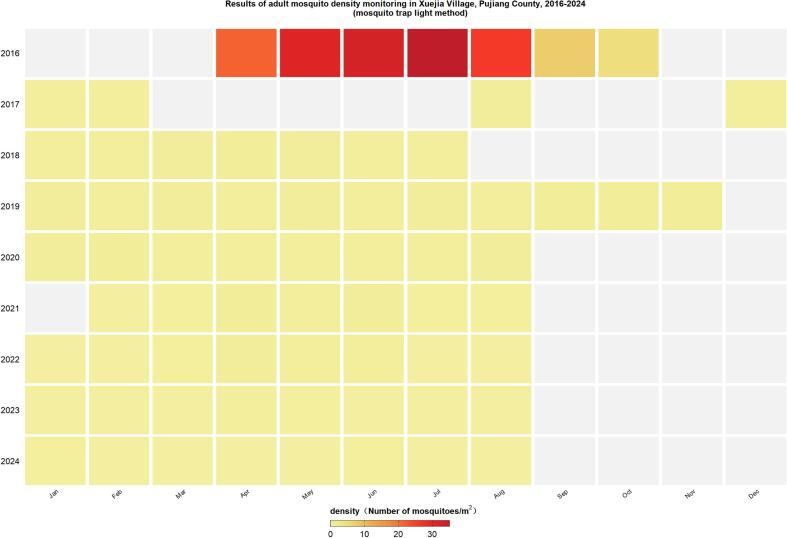
Results of adult mosquito density monitoring in Xuejia Village, Pujiang County, 2016–2023 (mosquito trap light method).

In order to implement the “OH” concept and vector sustainable bio-control strategy, explore the threshold forward in dengue fever prevention, in August 2016, Zhejiang province started from countryside villages, leading the “mosquito-free village” exploration research [18]. “Mosquito-free village”(or MVF) focus on “ecotope, vectors and health”, and implement mosquito-borne disease control measures at the grass-roots level in rural areas through building rural organizations, upgrading the environment and infrastructure, and at the same time carrying out biophysical prevention and control, popularizing health education among the villagers, and implementing comprehensive measures focusing on environmental management [19]. After more than half a year of efforts, the mosquito density in Xuejia village in 2017 was significantly lower than in the same period in 2016 [20]. Following health promotion and education, the knowledge rate and correct behavior formation of villagers against mosquitoes was 15 % higher than in contrast villages, and the knowledge and correct conduct formation rate of villages against mosquito in Xuejia Village was 82.80 %, significantly higher than that of contrast villagers (67.80 %) [19]. In addition, the villagers' willingness to fight mosquitoes has also been qualitatively improved. Adult mosquito densities in Xuejia Village remained at very low levels throughout 2017–2024 (Fig. 1).

(2) *Fly-Free village:*

In 2019, Hongxi Village of Tianning Town, Jiashan County, Jiaxing City in the northern plain of Zhejiang Province, Liujiaotang Village of Jindong District, Jinhua City in the central basin of Zhejiang Province, and Shangtianbanling Village of Sandu Town, Songyang County, Lishui City in the southern mountainous region of Zhejiang Province were selected to carry out a pilot study of “fly-free village” combined with garbage classification [21].

#### 2.4.3 Four Pest-Free village2.0——Mosquito and Fly-Free village:

On the basis of the research on mosquito-free and fly-free village, the research on the four pests-free village version 2.0:“mosquito-free and fly-free villages” [22] was carried out in 2020, and the fly-free and mosquito-free villages of Hongxi Village, Tianning Town, Jiashan County, Jiaxing City, and Yumin Village, Yaozhuang Town, combined fly-free and mosquito-free, and carried out unified environmental management and remodeling.

#### 2.4.4 Four Fest-Free village3.0——Four Pest-Free village:

Zhejiang Provincial Patriotic Health Office attaches great importance to countryside vectors health work, on the basis of the successful pilot construction of mosquito-free villages and fly-free villages and the good response from the whole province, it has formulated the indicator system of “Mosquito-free village”, “Fly-free village” and “Four Pests-free village” indicator system [23].

**Fig. 2:**
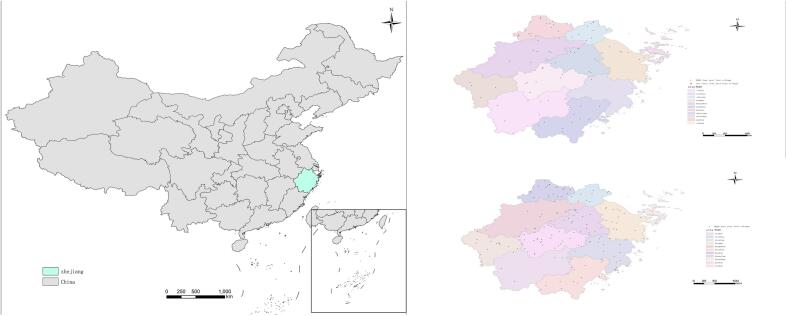
2021–2022 demonstration villages of four pest- free village in Zhejiang Province (Map Review Number:GS(2024) No.0650).

At the beginning of 2021, the Health and Wellness Commission of Zhejiang Province took the lead in carrying out the “construction of villages for the elimination of the four pests, with a focus on the elimination of mosquitoes and flies”, to further explore the strategy for the sustainable control of disease vector organisms in the countryside. At present, with Xuejia Village in Pujiang County, Jinhua City, Shenjiadun Village in Deqing County, Huzhou City, Hongxi Village in Tianning Town, Jiashan County, Jiaxing City, and Yumin Village in Yaozhuang Town as its representatives.The province has built 1000 “four pest-free villages”, of which 250 provincial model villages were built in 2021–2022, as shown below (Fig. 2).

#### 2.4.5 Four Fest-Free village4.0——Mosquito Free-Community and Campus:

In 2023, research was conducted in Jiaxing City and Pujiang County on “mosquito-free community [24]“in version 4.0 of the four pest-free village, and the creation of “mosquito-free campus” in conjunction with the Hangzhou Medical College, in which the use of mosquito-free gates received a favorable response.

The four pest-free village is an innovative concept and strategy of countryside sustainable vector management in Zhejiang province (ZJCSVM), which is in line with “OH”. It is an exploration of the path of sustainable vector control in the countryside of China [25]. The main measures for mosquito-free villages include an integrated selection of environmental modification, environmental management, and sustainable biological and physical control technical measures, such as irrigation management, paddy rotation, paddy fish farming [26], paddy loach farming [9], installation of mosquito lamps, mosquito gates, and sugar-baited mosquito traps. It is expected that under the background of the construction of beautiful China, the sustainable vector biological control strategy of Zhejiang, represented by mosquito-free villages, will rely on rural revitalisation, combine with the construction of beautiful villages and future villages, and incorporate health elements, so as to create a strong backing for the sustainable control of countryside vector organisms.

The construction of the four pest-free village from version 1.0 to 4.0 closely follows the two core elements and four pillars of the GVCR. The four pillars of action for the four pest-free villages are environmental clean-up, integrated measures combining physical and biological measures, health promotion and mosquito-borne disease control for both urban and rural residents.

**Fig. 3:**
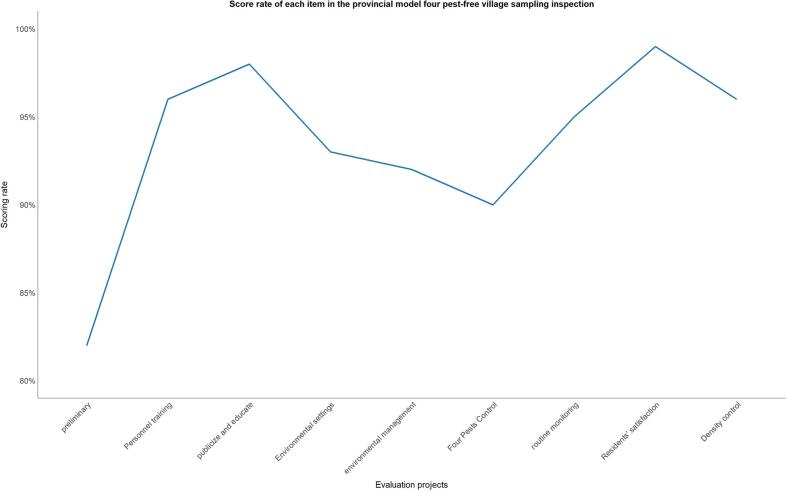
Score rate of each item in the provincial model four pest-free village sampling inspection.

The latest provincial model “four pest-free village” sampling scale shows that the “density control” section is the section with the lowest score (Fig. 3), so density control should be strengthened in the construction of pest-free villages in the future.

## 3 Discussion

On February 3, 2024, the Central Committee of the Communist Party of China (CPC) promulgated the State Council's Opinion on leveraging the ‘Triple Village Model and Welfare of All Villages’ project to effectively advance the comprehensive revitalization of rural areas, marking the 12th directive since the 18th National Congress dedicated to enhancing ‘Three Farmers’ initiatives [27]. In order to reduce the disturbance of human life by vectors, implement the “OH” concept, prevent and control major vector-borne diseases, Zhejiang Province has researching and exploring the practice of sustainable biological control of countryside vector. Zhejiang Province in the countryside sustainable vector management of organizational management, sustainable vector monitoring and control of technical support and guidance, sustainable human resources team building, sustainable beautiful countryside ecological environment construction and other aspects of active exploration, which is free of four pests in the village cost-effectiveness is significant, in the prevention and control of dengue fever is worth promoting [28]. The Patriot Health Campaign, which originated in the “Eliminate the four pests”, has been celebrating its 72nd anniversary, and the new era of the Patriot health campaign requires shortcuts and innovative approaches, recommending that IHWH accelerate the transformation and upgrading at all levels [29], and improve the environment in the countryside, eliminate media and hosts, and raise the health level of the people of China.

### 3.1 Lessons in prevention and control

The results of our four pest-free village have three key policy implications. First, strengthen inter- and intra-sectoral action and collaboration is necessary to make the construction of four pest-free villages more efficient or sustainable. Policy makers need to develop a standardized, legalized system in line with local needs and call for compliance by the participating sectors.

Second, the construction of four pest-free villages engages and mobilizes communities and improves the efficiency of the construction of four pest-free villages as well as the residents' knowledge of vectors and vector-borne diseases. These initiatives are consistent with the concept of engaging and mobilizing communities proposed by WHO. Policy makers need to develop targeted knowledge training and mobilization for different countries and regions, and for different residents, to promote the construction of four pest-free villages in Zhejiang, across the country and around the world, and to contribute to sustainable control of vectors and vector-borne diseases.

Third, Zhejiang Province's recent success in reducing the incidence and density of vector-borne diseases that continued expanding of the four pest-free villages is both reasonable and necessary. Developing countries that are affected by vector-borne diseases similar to China's could apply this model to their areas. Our construction of the four pest-free village illustrates that sustainable vector control in different regions according to the different needs of different regions and the different needs for the eradication of vector-borne diseases, especially mosquito-borne diseases, can protect the environment and save costs.

We realize that political factors may influence some recommendations. We have found signs of this influence in the construction of four pest-free villages. We recommend that policy support be strengthened when undertaking sustainable vector control. Other developing countries, especially those with similar experiences to China, should also be able to progressively control vectors to a very low level while protecting the environment.

## 4 Conclusion

We have carried out the four stages of the four pest-free village based on the OH concept and the GVCR proposed by WHO. The objective of the project is to build a new environmentally friendly community while reducing the density of several vectors to a manageable level. We found that the four pest-free villages are effective in controlling the incidence of vector-borne diseases, but need to make further enhancements in controlling vector densities. Without long-term, sustainable vector control, four pest-free villages cannot be maintained and promoted. We encourage developing countries and regions to build four pest-free villages and work together to promote further upgrading of four pest-free villages.

## CRediT authorship contribution statement

**Jing Ni:** Writing – review & editing, Writing – original draft, Software, Resources, Formal analysis, Data curation, Conceptualization. **Jinna Wang:** Validation, Supervision, Data curation. **Wenrong Zhang:** Writing – review & editing, Validation, Methodology, Data curation. **Enfu Chen:** Supervision, Project administration, Funding acquisition, Conceptualization. **Yi Gao:** Visualization, Supervision, Project administration, Conceptualization. **Jimin Sun:** Supervision, Resources, Project administration, Funding acquisition. **Wenzhong Huang:** Supervision, Investigation, Data curation. **Jun Xia:** Validation, Project administration, Funding acquisition. **Weiming Zeng:** Software, Resources, Methodology, Data curation. **Junxiang Guo:** Supervision, Project administration, Methodology, Funding acquisition. **Zhenyu Gong:** Writing – review & editing, Investigation, Data curation, Conceptualization.

## Funding

Supported by the Chinese Center for Disease Control and Prevention 2023 Pre-research Project on Health and Wellness Standardization in Public Health (BZ2023-Q039); Medical and Health Research Project of Zhejiang Province (2023KY638).

## Declaration of competing interest

The authors declare the following financial interests/personal relationships which may be considered as potential competing interests: Zhenyu Gong reports financial support was provided by Zhejiang Provincial Center for Disease Control and Prevention. Reports a relationship with that includes:. Has patent pending to. If there are other authors, they declare that they have no known competing financial interests or personal relationships that could have appeared to influence the work reported in this paper.

## Data Availability

Data will be made available on request.

## References

[1] WHO (2024). Launches global strategic plan to fight rising dengue and other Aedes-borne arboviral diseases. https://www.who.int/news/item/03-10-2024-who-launches-global-strategic-plan-to-fight-rising-dengue-and-other-aedes-borne-arboviral-diseases.

[2] WHO (2024). Global vector control response 2017–2030. https://www.who.int/publications-detail-redirect/9789241512978.

[3] Fite J., Baldet T., Ludwig A., Manguin S., Saegerman C., Simard F., Quénel P. (2025). A one health approach for integrated vector management monitoring and evaluation. One Health.

[4] Singh B., Kumar D., Kumar G., Saroha P., Vikram K., Gupta S.K., Singh H. (2024). Insecticidal paint: an alternate integrated vector management strategy for mosquito control. Process. Saf. Environ. Prot..

[5] Chanda E. (2020). Disregarding reservoirs of disease vectors: a surveillance paradox in Africa. EClinicalMedicine.

[6] Liew J.W.K., Selvarajoo S., Phang W.K., Mah Hassan M., Redzuan M.S., Selva Kumar S., de Silva J.R., Lau Y.L., Vythilingam I. (2021). Improved *Aedes*/dengue field surveillance using gravid oviposition sticky trap and dengue NS1 tests: epidemiological, entomological outcomes and community acceptance. Acta Trop..

[7] Sim S., Ng L.C., Lindsay S.W., Wilson A.L. (2020). A greener vision for vector control: the example of the Singapore dengue control programme. PLoS Negl. Trop. Dis..

[8] Zhejiang climate (2010). Climate overview of Zhejiang - Climate change. https://zj.weather.com.cn/qhbh/zjqh/12/1207225.shtml.

[9] Wu Y., Li T., Liu Q., Wang J., Luo M., Gong Z. (2025).

[10] General Administration of Quality Supervision, Inspection andQuarantine of the People’s Republic of China, Administration Standardization (2009). https://openstd.samr.gov.cn/bzgk/gb/newGbInfo?hcno=DE4E751FC4E05893E8B7A02DCEB826A5.

[11] General Administration of Quality Supervision, Inspection andQuarantine of the People’s Republic of China, Administration Standardization (2009). http://www.nhc.gov.cn/wjw/s9498/200908/42405.shtml.

[12] General Administration of Quality Supervision, Inspection andQuarantine of the People’s Republic of China, Administration Standardization (2009). http://www.cdc-gov.cn/newsview-727.html.

[13] General Administration of Quality Supervision, Inspection andQuarantine of the People’s Republic of China, Administration Standardization (2009). http://www.cdc-gov.cn/newsview-724.html.

[14] General Administration of Quality Supervision, Inspection and Quarantine of the People’s Republic of China, Administration Standardization (2009). http://www.nhc.gov.cn/wjw/s9498/202305/b52737b909244542a2496e7b624a69b2.shtml.

[15] General Administration of Quality Supervision, Inspection and Quarantine of the People’s Republic of China, Administration Standardization (2009). https://openstd.samr.gov.cn/bzgk/gb/newGbInfo?hcno=A5530916ADFD6981D4189F12C75A3752.

[16] General Administration of Quality Supervision, Inspection and Quarantine of the People’s Republic of China, Administration Standardization (2009). http://www.nhc.gov.cn/wjw/s9498/201207/55317.shtml.

[17] General Administration of Quality Supervision, Inspection andQuarantine of the People’s Republic of China, AdministrationStandardization (2009). http://www.cdc-gov.cn/newsview-731.html.

[18] Guo S., Huang W., Ling F., Wu H., Sun J., Lou Y., Gong Z., Hou juan, Chen E. (2018). Discussion on the construction standards and evaluation index system of ’ mosquito-free village ’. Chin. J. Vector Biol. Control.

[19] Chen E., Guo S., Huang W., Sun J., Gong Z. (2019). Evaluation index system for mosquito control and ’ mosquito-free village ’ construction in rural areas. PrevMed.

[20] Wu H., Liu Y., Huang W., Ling F., Lou Y., Sun J., Gong Z., Hou J., CHEN E. (2018). Evaluation on construction of “mosquito-free village” in Pujiang county, Zhejiang. Chin. J. Vector Biol. Control.

[21] Wang J., Gao Y., Hou J., Wang X., Wu Y., Liu Q., Li T., Zhang X., Gong Z. (2021). An exploration of standards and systematic assessment indices for “fly-free villages.”. Chin. J. Vector Biol. Control.

[22] Qi Y., Wang J., Wu Y., Fu X., Li Y., Huang J., Gong Z. (2021). Effect evaluation and measures of ’ mosquito-free village ’ construction in Hongxi Village, Jiashan County, Zhejiang Province. Dis. Surveill..

[23] Wang J., Wang X., Hou J., Guo J., Wu Y., Liu Q., Li T., Luo M., Gong Z. (2021). A discussion on the construction experience and standard of “four pests-free villages” in Zhejiang province, China. Chin. J. Vector Biol. Control.

[24] Guo S., Gong Z., Wu Y., Hou J., Wang J. (2019). Exploring indicators and levels of evaluation for sustainable community mosquito control. http://kns-cnki-net-s.webvpn.hmc.edu.cn:8118/kcms2/article/abstract?v=gisQO9UvOsZDDdoutV26-erqsvjPBkCG5-7_PucD8SjnJClPyAsE3RV8jTzjM9wzjUta--JAW7R6-FwraTzY74W5_3WyaWhmCnli54hSdy6seaV3FyQcuwjEXDEYKMETIbC6V4nJ5tr3LOMd1Ccn92TFtMgh2DswTTu_kWyCtp0U5gpoQIcL95M3RhP1G0qsaGWmj-l67E_5Kq9_pv3mUw==&uniplatform=NZKPT&language=CHS.

[25] Liu Q., Meng F., Lu L., Wang C. (2006). Research on sustainable vector control in China. Chin. J. Vector Biol. Control.

[26] Zhao T., Xue R.-D. (2024). Vector biology and integrated Management of Malaria Vectors in China. Annu. Rev. Entomol..

[27] Zhu Y. Opinions of the Central Committee of the Communist Party of China and the State Council on Learning and Applying the Experience of the “Thousand Villages Demonstration and Ten Thousand Villages Improvement” Project to Powerfully and Effectively Promote the Comprehensive Revitalisation of Rural Areas_Central Document. https://www.gov.cn/zhengce/202402/content_6929934.htm.

[28] Guo S., Huang W., Sun J., Wu H., Liu Y., Zhang Y. (2024). Long-term effectiveness evaluation of the construction of “mosquito-free village” in Pujiang County. Prev. Med..

[29] Gong Z., Gao Y., Mao Y., Wang X. (2022). The normalization of COVID-19 epidemic prevention and control calls for patriotic health campaign inherit and innovation. Dis. Surveill..

